# A Step-by-Step Protocol for Optogenetic Kindling

**DOI:** 10.3389/fncir.2020.00003

**Published:** 2020-01-29

**Authors:** Elvis Cela, P. Jesper Sjöström

**Affiliations:** ^1^Brain Repair and Integrative Neuroscience Program, Centre for Research in Neuroscience, Department of Medicine, Department of Neurology and Neurosurgery, Montreal General Hospital, The Research Institute of the McGill University Health Centre, Montreal, QC, Canada; ^2^Integrated Program in Neuroscience, McGill University, Montreal, QC, Canada

**Keywords:** epilepsy, optogenetics, seizure, animal model, kindling, protocol, Channelrhodopsin

## Abstract

Electrical kindling, repeated brain stimulation eventually resulting in seizures, is widely used as an animal model of epileptogenesis and epilepsy. However, the stimulation electrode used for electric kindling targets unknown neuronal populations and may introduce tissue damage and inflammation. Optogenetics can be used to circumvent these shortcomings by permitting millisecond control of activity in genetically defined neurons without gross injury or inflammation. Here we describe an easy step-by-step protocol for optogenetic kindling – optokindling – by which seizures are eventually elicited in initially healthy mice through repeated light stimulation of neurons expressing Channelrhodopsin-2 (ChR2). Chronic EEG recordings may be performed over large time scales to monitor activity while video camera monitoring may be used to assess the behavioral severity of seizures. In conclusion, with optokindling, neuroscientists can elucidate the circuit changes that underpin epilepsy while minimizing the contribution of confounding factors such as brain damage and inflammation.

## Introduction

Epilepsy is the fourth most common neurological disorder with over 3 million people affected in the United States alone (Hirtz et al., 2007). It is characterized by recurring seizures, or aberrant neuronal firing, and epileptogenesis is the process by which the healthy brain becomes epileptic (Chang and Lowenstein, 2003). The steps that underpin epileptogenesis remain unclear and they require animal models for their elucidation (Löscher, 2002). Animal models of induced seizures such as electrical kindling have been particularly helpful as they can transform a healthy animal into an epileptic one gradually over time while parameters such as seizure severity and duration are monitored (Goddard, 1967). However, with electrical kindling, it is not possible to target specific neuronal types during stimulation. Furthermore, the stimulating electrode introduces marked tissue damage, making it hard to tease apart the contribution of aberrant activity to epileptogenesis from that of brain damage and inflammation. This lack of experimental control has hampered progress toward a complete understanding of epileptogenesis at the neuronal level.

By using optogenetics, it is possible to circumvent these drawbacks by allowing genetic labeling and activity control of specific subsets of neurons. Indeed, optogenetics has been used in several studies to halt, as well as elicit seizures (Cela and Sjöström, 2019). Recently, we developed an optogenetic variant of classical electrical kindling (optokindling) based on repeated laser-light stimulation of pyramidal cells (PCs) in motor cortex (M1) (Cela et al., 2019). Our optokindling model mimicked classical electrical kindling in several ways: increased seizure severity and increased seizure duration over stimulating sessions, decreased seizure threshold and long-term retention of seizure susceptibility (Cela et al., 2019). Since we fluorescently tagged opsin-expressing PCs, we could identify the neurons that were directly stimulated. Furthermore, astrogliosis and gross neuronal damage were undetectable in our model, because the fiberoptic patch cable used for laser-light delivery did not penetrate the brain but was implanted above it. Our optokindling protocol resulted in 9 out of 12 animals developing seizures within 13 sessions of stimulation (Cela et al., 2019).

Here, we describe in detail and in simple terms the necessary steps to carry out optokindling in initially healthy wild-type mice. The present optokindling protocol can easily be adapted for use with other cells types.

## Survival Surgery

### Virus Handling and Storage

The below steps outline how to obtain, prepare, and use the virus for opsin delivery.

*Note 1:* Plasmids for custom viral constructs can be ordered from Addgene. The choice of promoter will determine efficiency and timecourse of expression, as well as the cell specificity.

*Note 2:* Adeno-associated virus (AAV) vectors can be ordered from university virus cores (e.g., Pennsylvania or North Carolina). Several different serotypes of virus have been used to express ChR2, from AAV2 to AAV9. In our study, we decided to use AAV5 after trying AAV2 and AAV8 with mixed success. The choice of virus serotype is not always straightforward, as outcome may depend on many factors, such as cell type, tissue, species, animal age, etc. In particular, different serotypes preferentially infect different tissue, a concept known as tropism (Naso et al., 2017). Viral injection can also result in an immune response, such that subsequent injections with the same serotype may be considerably less efficacious; this problem can be alleviated by switching serotype (Mendoza et al., 2017). Previous discussion of genetic targeting techniques for opsins should guide decision making with regard to serotype (Sjulson et al., 2016; Cela and Sjöström, 2019). Ultimately, the choice of serotype is, however, largely a matter of trial and error.

(1) To aliquot the virus, ensure proper protection is worn in accordance with the chosen biosafety level of the virus to be used.
(2) Thaw viral stock on ice outside of biosafety cabinet and aliquot 5 μL into small Eppendorf tubes for use on surgery day.
(3) Discard waste into 10% bleach solution.
(4) Label and store aliquots at −80°C.

### Stereotaxic Surgery for Viral Expression of ChR2 and Ferrule Implantation

The protocol presented here outlines how to use optokindling to gradually induce seizures in initially healthy animals.

**FIGURE 1:**
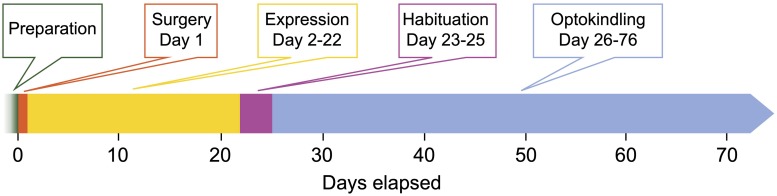
Timeline of the optokindling protocol. This timeline indicates the number of days elapsed from start as well as the duration of each step. *Preparation:* All materials for surgery and optoelectronic components are prepared. *Surgery (1 day):* Animals are injected with AAVs and implanted with fiberoptic ferrules and EEG electrodes. This important step sets the foundation for the rest of the experiments. *Expression (21 days):* This waiting period is required to reach sufficiently high ChR2 expression levels. *Habituation (3 days):* To ensure that animals are not stressed by experimenter handling during the subsequent optokindling period, animals are habituated. *Optokindling (50 days):* While optokindling, animal behavior is monitored, and outcomes such as seizure duration and severity are quantified. The time required for optokindling is 50 days provided the 25 stimulation sessions are spaced ∼48 h apart. It is likely possible to stimulate more often, e.g., every 24 h.

*Note:* Prior to survival surgery, all surgical tools should have been autoclaved and ferrules for implantation prepared (Preparation stage, Figure 1). It may be important to use only male mice to avoid hormonal contributions to seizure outcome, unless this is what is studied (Curry, 1974). We furthermore recommend using animals in the P30-45 age range – younger animals have critical periods that may affect epileptogenesis, while older animals are not ideal should, e.g., acute slice experiments subsequently be required. We recommend using control animals where AAV-EYFP is injected instead of AAV-ChR2, to test for effects of the surgical procedure itself. Another type of control is to inject the AAV-ChR2 virus but ommit the optokindling.

*Preparation:* Confirm that you will have the right number of animals required for surgery.

(1) Make sure fiber-optic ferrules have been prepared according to standard procedures (Zhang et al., 2010). The length of fiber protruding from the ferrule should be sufficient to target the brain area of interest.
(2) Using a micropipette puller (e.g., Sutter P-1000, Narishige PC-10, or Zeitz DMZ), pull glass micropipettes with ∼100 μm or smaller tip size for viral injection. Alternatively, small-gauge Hamilton syringes (e.g., Neuros series) can be used.
(3) Prepare working concentrations of Meloxicam (20 mg/kg) and Buprenorphine (0.1 mg/kg).
(4) Prepare EEG recording screws by soldering copper wire to stainless steel screws (e.g., McMaster Carr, 000–120 × 1/8).

#### Day 1

*Note*: Keep the surgeries as short as possible (e.g., under 3 h). Lengthy surgeries typically result in lower success rates. Follow standard analgesia/anesthesia practises according to your local Standard Operating Procedure (SOP). The volume of AAV injected depends on serotype and desired volume of brain area to target. We used 1.2 μl per hemisphere of the original titer provided by the UNC virus core, which was 1−8×10^12^ viral genomes per milliliter. We recommend starting with the full concentration of virus and performing serial dilutions in 10× increments until the desired volume of transfection is reached after imaging. We induced anesthesia using an isoflurane/oxygen mix (4%) while the animal is placed in a scavenger cage (e.g., Harvard Apparatus, 75-0239). After the animal is non-responsive to tail-pinch, quickly remove the animal from the cage and connect to the nose hole of the stereotaxic holder (e.g., Stoelting Just for Mouse). This ensures that the animal is sufficiently anesthetized.

(1) Once in the stereotax, affix the ear bars and position the head securely so that the head does not move when lightly prodded. Keep an eye on animal breathing rate throughout the surgery, to make sure that rate of respiration does not depress severely.
(2) Shave the hairs on the head using a trimmer (e.g., Harvard Apparatus, 729063) and apply iodine solution to the head over the head. Make a midline incision on the head using a scalpel so that the skull is visible. Wipe off any blood with cotton swabs and then peel back any fatty tissue obstructing the full view of the skull.
(3) Mark the skull locations undergoing injection using a surgical marker over the *X* and *Y* stereotaxic coordinates obtained from the Mouse Atlas (e.g., Paxinos and Franklin AP). Open a 1-mm craniotomy over the left target brain using a 9-mm dental drill (e.g., Ram Products, TECH2000). Hydrogen peroxide wash may help stop blood emanating from the skull prior to craniotomy.
(4) Using a programmable pump (e.g., Harvard Apparatus PHD series), inject 1.2 μL AAV-ChR2 bilaterally into brain area under investigation. Aim for 100 μl/min allowing for an additional 5 min for the injection needle to stay in place to help the virus diffuse.
(5) If performing bilateral injections, follow the same procedure outlined in (4) on the other hemisphere.
(6) Dry the skull thoroughly (e.g., Puritan cotton-tipped applicators) whilst making sure to keep the craniotomy area moist with saline solution.
(7) Using the stereotax, place the ferrule with optic fiber attached that was made in Preparation day to the desired brain depth (pia if stimulating cortex). Begin making a mound around the ferrule using dental cement (e.g., Patterson Dental Ortho-Jet). If more stability is required, warmed agar can be used as well, although make sure it has cooled to 37°C before placing it down.
(8) If performing bilateral stimulation, follow the same steps as in (7) with the other ferrule.
(9) Using the dental drill, make holes that are just to the depth of the pia but no further – this is where the electrode screws will be affixed to the skull. The recording screws should lie as close as possible to the stimulation site. The ground reference screws can be placed close to the cerebellum, one on each hemisphere for bilateral recording.
(10) After the recording and reference screws have been affixed to the skull, continue building up the dental cement mound that was started in step (6).
(11) Remove the animal from the stereotax and allow recovery on a heating pad.
(12) Rinse the glass micropipette with 10% bleach and discard in a sharps container.
(13) Discard excess virus in 10% bleach and disinfect all surfaces and instruments that may have come in contact with the virus. Re-freeze unused virus at −80°C.
(14) When the animal has awakened, place it back in the animal rack cage.

#### Day 2

(1) Refer to your university SOPs for pain assessment and management. Inject analgesics such as Meloxicam according to the SOP.
(2) Check on the animals at least once a day for the next week to make sure they recover from surgery properly.

### Optokindling Procedure

*Note:* Waiting 21 days is recommended for most AAVs to sufficiently drive expression of ChR2 (Figure 1). Depending on the vector you use, you may have to wait a different length of time.

#### Day 23

(1) Habituate the animals for the next three days by connecting them to optic fibers (e.g., Thorlabs FT200EMT), allowing ∼20 min inside the recording cage once a day.
(2) Check light responses by stimulating with two pulses of light 10 ms in duration while monitoring the amplified EEG signal (e.g., AM Systems Extracellular Amplifier). Responses in the EEG may vary from hundreds of microvolts to several millivolts depending on extent of viral expression and light stimulation intensity, as well as ChR2 variant used.

#### Day 26

**FIGURE 2:**
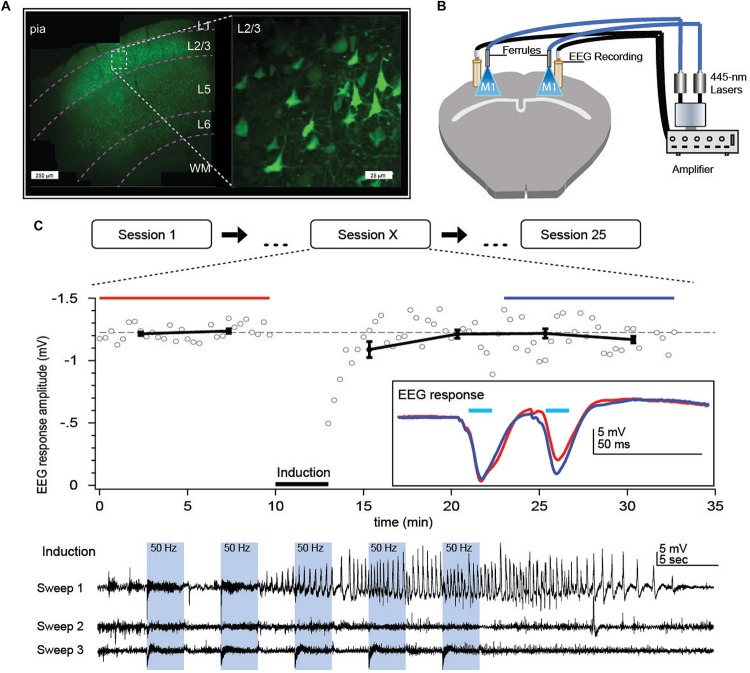
Optokindling protocol for gradual increase of seizure susceptibility *in vivo.* **(A)** Coronal M1 section indicating ChR2 expression primarily in L2/3. Inset shows close-up of L2/3 ChR2-expressing PCs. **(B)** Bilateral implantation of recording screws allows EEG recording whilst fiber-optic ferrule implantation above pia facilitate ChR2 activation without damaging the brain. Fiber optic cables were air-coupled to 445-nm lasers. EEG signals were amplified and then digitized by a computer (not shown). **(C)** During each stimulation session, M1 was repeatedly exposed to 445-nm laser light (“Induction”), delivered as 15 bouts of 3-s-long 50-Hz bursts of 5-ms pulses, divided into three sweeps delivered once a minute. Sessions were repeated every 2 days, 25 times or more. In this example, a prominent seizure was evoked in the first induction sweep of session *X* = 15. We measured EEG responses to 30-Hz paired-pulse laser stimuli for 10 min before and 20 min after the optokindling induction to look for long-term changes in circuit plasticity. *Inset:* Paired-pulse EEG responses before (red) and after (blue) indicated a change in EEG dynamics but not amplitude. Reproduced from Cela et al. (2019).

*Note:* Try using two cameras, one above and one to the side of the animal for improved capture of behavior (Figure 3). Relying on two or more people to score seizure behavior helps to eliminate scoring bias, resulting in more reliable results.

(1) Run stimulation protocol consisting of two pulses at 10 ms repeated every 20 s for 10 min, then 50-Hz bouts for three times, followed by two pulses at 10 ms repeated every 20 s for 20 min again (Cela et al., 2019). The total stimulation time will be ∼33 min.
(2) At the start of the EEG recording, also start video recording to capture behavioral severity of seizures (e.g., Logitech C525 webcam). Later, the video will be used to assign a Racine score to behaviorally quantify seizures.
(3) Return the animal to its original cage after unhooking ferrule and EEG attachments.
(4) Repeat these stimulation sessions every 48 h until at least session 25 has been reached (Figure 2).

**FIGURE 3:**
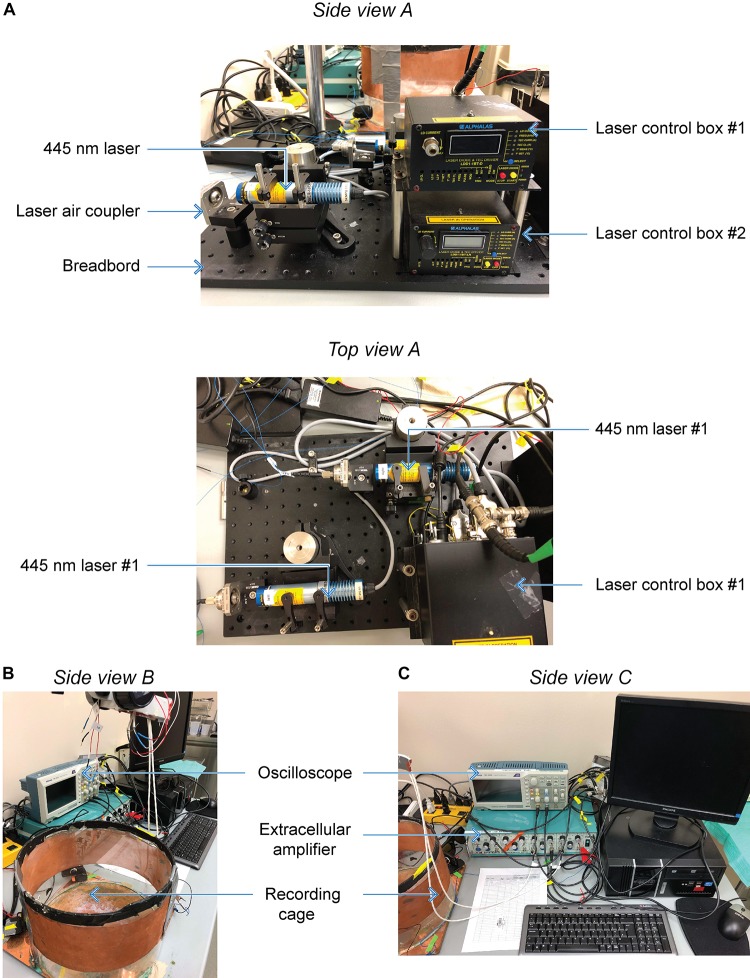
Custom optokindling platform for simultaneous ChR2 excitation and EEG recording. **(A)** Side view A shows both laser control boxes with one 445-nm laser mounted to a breadboard. The laser air coupler collects the laser beam into an FC-PC fiber optic cable using a fiber port collimator. Top view A shows both 445-nm lasers mounted on the breadboard. **(B)** Side view B shows the Faraday cage where EEG recordings are performed as well as the extracellular amplifier used during recordings. **(C)** Side view C shows the oscilloscope used to display EEG signals during acquisition as they are digitized by the data acquisition board and stored on the computer.

## Materials and Reagents

### Hardware

Computer (SuperLogics, SL-DK-H61MX-1D).
Data acquisition board (e.g., NI PCI-6221 or newer PCIe DAQ).
BNC cables (Newark).
Data acquisition software (Igor Pro, Wavemetrics Inc.).
Glass pipette puller (Sutter P1000).
Copper sheet (McMaster Carr, 89675K31).
Extracellular amplifier (AM Systems, Model 1700).
Recording camera (Logitech, C525).
Recording software (ISpy).

### Optics

ChR2 excitation laser (e.g., inexpensive eBay laser, or Alphalas GmbH, Monopower-455-150-MM-TEC).
FC-PC patch cable (Thorlabs, M83L01).
FC-PC fiber port collimator (Thorlabs PAF2P-18A).
Breadboard (e.g., Thorlabs, MBH1224).
V-clamp (e.g., Thorlabs, C1513/M).
Ceramic ferrules (Thorlabs, CFLC230-10).
Fiber stripping tool (Thorlabs, T10S13).
Three-hole fiber stripper (Thorlabs, FTS4).
Fiber polishing film (Thorlabs, LF1D, LF3D, LF6D).
Fiber polishing disk (Thorlabs, D50-FC).

### Surgery

Channelrhodopsin-2 Adeno-associated virus (e.g., UNC Virus Core).
Dental cement powder (Patterson Dental, 459-8371).
Dental cement liquid (Patterson Dental, 459-3869).
Bulldog clamps (Fine Science Tools, 18050-28).
Silicone mixing cup (Henry Schein, 5100062).
Betadine solution (Purdue Pharma, 40889).
Absorption sponges (Fine Science Tools, 18105-03).
Cauterizer (Fine Science Tools, 18010-00).
Skin marker (Fine Science Tools, 18000-30).
Extra fine Forceps (Fine Science Tools, 11152-10).
Bonn scissors (Fine Science Tools, 14084-08).
Dumont forceps (Fine Science Tools, 11251-35).
Straight forceps (Fine Science Tools, 11000-12).
Scalpel handle (Fine Science Tools, 10003-12).
Scalpel blades (Fine Science Tools, 10010-00).
Suture needles (Fine Science Tools, 12050-01).
Microdrill burrs (Fine Science Tools, 19008-14).
Cordless hair clipper (Harvard Apparatus, 729063).
Stereotax (e.g., Just for Mouse Stoelting Co., 51730).
Mouse mask (Harvard Apparatus, 72-6044).
Syringe pump (Harvard Apparatus, 70-4507).
Anesthesia system (Harvard Apparatus, 75-0239).
Heating pad (Kent Scientific, DCT-15).
Microdrill (Ram Products, TECH2000ON/OFF).
Lubricant eye ointment (AKORN, Artificial Tears).
Mouse atlas (Paxinos and Franklin, Academic Press).
Stereomicroscope (Leica A60).
Meloxicam (Metacam, 5 mg/ml).
Buprenorphine (Vetergesic, 0.3 mg/ml).
EEG recording screws 000–120 × 1/8 (McMaster-Carr, 90910A600).
Gold-plated jacks (Warner Instruments, 64-132).

## Summary and Future Directions

Optokindling shares several hallmark features with electrical kindling (Goddard, 1983), including the gradual development of a lowered seizure threshold and longer seizure duration, as well as an increase in behavioral severity of seizures over time (Cela et al., 2019). This increased propensity for more severe seizures of longer duration is retained in the long term even in the absence of ongoing stimulation, which as in Goddard’s original model (Goddard, 1967) is in keeping with a form of long-term memory, in further agreement with classic electric kindling.

By relying on gradual development of seizures based on cell-type specific activation, the optokindling model opens up new avenues for exploring the microcircuit plasticity that underlies epileptogenesis (Cela and Sjöström, 2019). This sets the optokindling model apart from other optogenetic models of epilepsy that rely on directly driving the seizures already in naïve animals (Khoshkhoo et al., 2017). In our hands, optokindling did not result in any detectable spontaneous seizures, as has been previously shown with electrical kindling (e.g., Michael et al., 1998). This is arguably both a pro and a con: it provides improved experimental control, but it comes at the cost of reduced biological realism, since epilepsy by definition involves spontaneous seizures. Additionally, we have highlighted typical setbacks associated with the protocol and how to circumvent them (Table 1).

**TABLE 1:** Troubleshooting – list of common pitfalls and how to circumvent them.

**Problem**	**Potential cause**	**Solution**
No EEG response during habituation	Virus leakage during injection or wrong serotype/promoter for cells of interest	(1) Slice brain of animal and visualize under microscope to see if virus is expressed to sufficient levels (2) Confirm placement of screws and electrical continuity
Cap falls off between stimulation sessions	Insufficient drying time between dental cement applications	Ensure that dental acrylic is put on in layers and allow drying between them
Animal is visibly uncomfortable during stimulation sessions	Animal is not sufficiently habituated to new housing or recording setup	(1) Make sure mouse is habituated properly and has had time to settle for 72 h if it is being shipped from elsewhere (2) Make sure mouse is habituated to recording setup for 3 days
Ferrule comes off between sessions	Animal is moving excessively during connection or insufficient levels of dental cement applied	(1) Using Dremel, slightly buff the bottom part of the ferrule that is being placed in the brain for better acrylic adherence (2) Make sure that only about half of ferrule is embedded to allow enough surface area for the sleeve mated to the optic fiber to hold on to
Insufficient power reaches end of ferrule	Ferrule is damaged during connection or laser is underpowered	(1) Make sure that FC-PC coupler has correct NA so that laser light is collected (2) Check for light leaks and/or breaks in the fiber ferrule
Mouse is gasping or wet cough during surgery	Surgery is >3 h	(1) Atropine can help loosen up airways (2) Try repositioning mouse in stereotax to open up trachea

Optokindling could also be improved upon. For example, optokindling may be combined with other readouts, such as calcium imaging, to take advantage of the genetic specificity (Khoshkhoo et al., 2017). While motor cortex was targeted in our original study (Cela and Sjöström, 2019), other brain areas such as hippocampus or amygdala are likely more susecptible (Goddard et al., 1969), allowing for more rapid optokindling. Indeed, a recent study shows that optogenetic kindling works well in the hippocampus (Klorig et al., 2019).

Using optokindling, seizures can be elicited by avoiding gross brain damage and targeting other cell populations of interest such as interneurons. The optokindling model may thus provide novel therapeutic insights not only for treatment of the symptoms of epilepsy, i.e., the seizures, but also for the actual process of epileptogenesis, which is causally related to chronic epilepsy (Cela and Sjöström, 2019).

## Data Availability Statement

The raw data supporting the conclusions of this article will be made available by the authors, without undue reservation, to any qualified researcher.

## Ethics Statement

The animal study was reviewed and approved by the Montreal General Hospital Facility Animal Care Committee (The MGH FACC), and adhered to the guidelines of the Canadian Council on Animal Care (CCAC).

## Author Contributions

All authors listed have made a substantial, direct and intellectual contribution to the work, and approved it for publication.

## Conflict of Interest

The authors declare that the research was conducted in the absence of any commercial or financial relationships that could be construed as a potential conflict of interest.
